# Genetic characterization of 11 microsatellite loci in Egyptian pigeons (*Columba livia domestica*) and their cross-species amplification in other Columbidae populations

**DOI:** 10.14202/vetworld.2018.497-505

**Published:** 2018-04-17

**Authors:** Sherif Ramadan, Ahmed Dawod, Osama El-Garhy, Amira M. Nowier, Marwa Eltanany, Miho Inoue-Murayama

**Affiliations:** 1 Wildlife Research Center, Kyoto University, Kyoto, Japan; 2 Department of Animal Wealth Development, Faculty of Veterinary Medicine, Benha University, Toukh, Egypt; 3 Department of Husbandry and Animal Wealth Development, Faculty of Veterinary Medicine, Sadat City University, Egypt; 4 Animal Production Department, Faculty of Agriculture, Benha University, Toukh, Egypt; 5 Department of Biotechnology Research, Animal Production Research Institute, ARC, Dokki, Egypt; 6 Department of Animal Wealth Development, Faculty of Veterinary Medicine, Benha University, Toukh, Egypt; 7 Wildlife Genome Collaborative Research Group, National Institute for Environmental Studies, Tsukuba, Japan

**Keywords:** Egyptian breed, genetic diversity, microsatellite, pigeon

## Abstract

**Aim:**

This study aimed to analyze the genetic diversity and relationships of 10 Egyptian pigeon populations belonging to *Columba livia domestica* speciesusing 11 microsatellite markers and to investigate the success of these markers amplification across another eight pigeon species.

**Methods:**

Genomic DNA was isolated from feather samples of179 pigeon samples from 10 Egyptian breeds: Asfer Weraq (n=14), Austoraly (n=20), Reehani (n=21), Messawed (n=17), Nemssawy (n=27), Otatti (n=12), Morasla (n=17), Tumbler (n=22), Halaby Asfer (n=10), and Karakandy (n=19) in addition to Japanese feral pigeons (n=30). Genotyping was done using 11 specific polymorphic microsatellite makers. Moreover, 37 samples not belonging to *C. livia domestica* but belonging to another eight pigeon species were genotyped. The polymerase chain reaction (PCR) products were electrophoresed on an ABI 3130xl DNA Sequencer. The basic measures of genetic diversity and phylogenetic trees were computed using bioinformatics software.

**Results:**

Across the 10 studied Egyptian populations, the number of alleles per locus ranged from 3 to 19 and the average number of alleles observed was 9.091. The lowest value of expected heterozygosity (0.373) was obtained for the Reehani breed, and the highest value (0.706) was found for Morasla breed. The overall expected heterozygosity of Egyptian pigeons was 0.548. The *F_ST_* coefficient which indicates fixation coefficients of subpopulations within the total population for the 11 loci varied from 0.318 to 0.114 with a relatively high mean (0.226). In our study, the *F_IS_* showed a relatively high average(0.037). The pairwise Reynolds’s genetic distance between the11 studied pigeon populations recorded lower values between Otatti and Austoraly (0.025) and between Morasla and Japanese feral pigeons (0.054). These results are supported by clustering pattern either by the neighbor-joining phylogenetic tree or by a Bayesian clustering of STRUCTURE with the admixture method.

**Conclusions:**

We confirm the applicability of the *CliµD17, CliµT17, CliµD16, CliµD32, CliµT13*, *CliµD01*, *PG1, PG2, PG4, PG6*, and *PG7* microsatellite markers among Egyptian domestic pigeons and across other pigeon species using cross-species amplification method. The information from this study should be useful for genetic characterization and for developing conservation programs of this important species.

## Introduction

Assessment of farm animal genetic diversity is important to identify native populations useful for food security and rural development. It allows breeders to identify, select, and develop new breeds in response to the changeable conditions such as climatic change, disease threats, human nutritional requirements, and changing market needs [1,2]. Native livestock breeds are a valuable source of genetic material because of their adaptation to harsh conditions and their tolerance to a range of diseases. Genetic and phenotypic characterization of animal genetic resources helps us for the development of more efficient production systems and breeding programs [3]. Improvement of production can be attained through mating of diverged populations which can result in hybrid vigor [4,5].

Darwin wrote about the high degree of phenotypic variation among domestic pigeons and their differences from their wild ancestors. Pigeon is considered as a model organism because it is easy to breed and study in the laboratory; it helped Darwin to understand how evolution works in general [6]. Pigeon breeding is a popular hobby worldwide, and over 350 different breeds are currently recognized. Domestication of pigeons involved intensive directional selection for a particular trait, followed by stabilizing selection; some pigeon breeds are under intensive selection for flight characteristics such as racing homers and others are bred for vocal and morphological traits differences [7]. Pigeons are bred for different purposes such as meat in the form of squabs, ornamental and show, and flying and racing competitions [8] and finally for experiments of cognitive sciences [9]. The 10 Egyptian native indigenous pigeon breeds used in this study do not belong to feral pigeons. Eight of these breeds: Asfer Weraq, Austoraly, Reehani, Messawed, Otatti, Morasla, Halaby Asfer, and Karakandy are characterized by strong homing and flying abilities and mainly used for certain kind of a very popular flying game in Egypt [10], whereas the last two (Nemssawy and Tumbler) are used for ornamental and show purposes. In Egypt, despite the importance of this species, researches on the genetic variation and relationship of local pigeon breeds are scanty [10].

The microsatellite is a marker of choice for studying the genetic diversity and relationships among closely related livestock breeds [11,12]. MtDNA of pigeon was used to construct a phylogenic tree for *C. livia*, *Streptopelia*, and other related taxa [13]. The cross-species amplification of marker sets previously developed for closely related species is considered as an alternative way than *de novo* marker development. Some early studies have shown the applicability of microsatellite markers among closely related species using cross-species amplification [14,15].

This study aimed to analyze the genetic diversity and relationships of 10 Egyptian pigeon populations (Asfer Weraq, Austoraly, Reehani, Messawed, Nemssawy, Otatti, Morasla, Tumbler, Halaby Asfer, and Karakandy) using 11 microsatellite markers. Also, to investigate the success of these markers’ amplification across other pigeon species such as Oriental turtle dove, White-bellied green, Emerald dove, Whistling green pigeon, Blue-crowned pigeon, Japanese wood pigeon, Victoria crowned pigeon, and Pied imperial pigeon. Such information can be useful to develop a sustainable genetic improvement and conservation programs for this valuable species.

## Materials and Methods

### Ethical approval

All aspects of the study were performed according to theguidelines established by the Ministry of Education, Culture,Sports, Science, and Technology in Japan (Notice No. 71). The protocol was approved by the Committee on the Ethics of Animal Experiments of the Wildlife Research Center of Kyoto University (Permit No. WRC-2017-002A).

### Sample collection and DNA extraction

A total of 179 pigeon feather samples from 10 Egyptian local breeds: Asfer Weraq (n=14), Austoraly (n=20), Reehani (n=21), Messawed (n=17), Nemssawy (n=27), Otatti (n=12), Morasla (n=17), Tumbler (n=22), Halaby Asfer (n=10), and Karakandy (n=19) in addition to Japanese feral pigeons (n=30) were obtained. Moreover, 37 feather samples were collected from eight wild pigeon species as shown in Table-1. Egyptian samples were collected from nine breeders in five provinces (Giza, Cairo, Kaliobia, Menofia, and Zagazig) located in the Nile river delta in the northern part of Egypt, whereas samples of Japanese feral pigeons and other wild species were collected from Osaka Museum of Natural History, Osaka, Japan, and from Rescue Center of Kyoto City Zoo, Kyoto, Japan. DNA was extracted from feather samples using the QIAGEN DNeasy Tissue Kit (QIAGEN, Valencia, CA, USA).

**Table-1:** Number of samples for domestic and wild pigeon species.

Domestic pigeon breed (*Columba livia domestica)*	n	Wild pigeon species	n
Asfer Weraq	14	Oriental turtle dove *(Streptopelia orientalis)*	15
Austoraly	20	White-bellied green *(Treron sieboldii)*	9
Reehani	21		4
Messawed	17	Whistling green pigeon (*Treron formosae)*	3
Nemssawy Asfer	27	Blue crowned pigeon (*Goura cristata*)	2
Otatti	12	Japanese wood pigeon (*Columba janthina janthina)*	2
Morasla	17	Victoria crowned pigeon (*Goura victoria)*	1
Egyptian Tumbler	22	Pied imperial pigeon (*Ducula bicolor*)	1
Halaby Asfer	10		
Karakandy	19		
Japanese feral pigeon	30		
Total	209	Total	37

### Mitochondrial cytochrome c oxidase subunit I gene (COI) analysis

Mitochondrial *COI* sequence was analyzed for one sample of the 10 Egyptian breeds and of Japanese feral pigeons. Moreover, one sample was sequenced from the four wild pigeon species: Emerald dove (*Chalcophaps indica*), Oriental turtle dove (*Streptopelia orientalis*), Whistling green pigeon (*Treron formosae*), and white-bellied green pigeon (*Treron sieboldii*) for comparison. The primers and polymerase chain reaction (PCR) condition are the same as described by Ramadan *et al*. [10].

The amplified products were purified using PCR Purification Kit (Roche, Mannheim, Germany), and the resultant products were sequenced using the same primers and the Big Dye Terminator ver. 3.1 Cycle Sequencing Kit (Applied Biosystems, Foster City, CA, USA) according to the standard protocol and electrophoresed on an ABI PRISM 3130xl sequencer (Applied Biosystems). The MEGA 6 Software (https://www.megasoftware.net) [16] was used for sequences alignment and to infer the phylogenetic relationships based on neighbor-joining [17] methods [18].

### Microsatellite analysis

Eleven labeled microsatellite markers (*CliµD17, CliµT17, CliµD16, CliµD32, CliµT13*, and *CliµD01* from the study of Traxler *et al*. [19] and *PG1, PG2, PG4, PG6*, and *PG7* from the study of Lee *et al*. [20]) were used in two multiplex PCR reactions employing the QIAGEN Multiplex PCR Kit (QIAGEN, Valencia, CA, USA). PCR conditions are the same as described by Ramadan *et al*. [10]. The PCR products were electrophoresed on an ABI 3130xl DNA Sequencer (Applied Biosystems), and the sizes of the fragments were estimated based on 400 HD Rox size marker using the GENEMAPPER software (Applied Biosystems, Foster City, CA, USA).

### Data analysis

Genetic diversity was assessed by calculating the observed and effective number of alleles (*N_A_* and *N_e_*) and observed and expected heterozygosity (*H_O_, H_E_*) using GENALEX version 6.0 [21]. Polymorphic information content (*PIC*) was calculated using CERVUS version 3 software [22]. *F*-statistics (*F_IS_*, *F_ST_*, and *F_IT_*) in addition to pairwise *F_ST_* [17] across the 11 studied populations were calculated using the GENEPOP version 3.4 [23]. Genetic distances among the 11 populations were evaluated by Reynolds’ genetic distance [24]. A phylogenetic tree was constructed based on the Reynolds’ genetic distance using the neighbor-joining [17] method [18]. The robustness of tree topologies was evaluated with a bootstrap test of 1000 resampling across loci. These processes were conducted using POPULATIONS version 1.2.30 software [25]. We investigated the clustering, and genetic structure of the 11 studied pigeon populations using STRUCTURE software [26]. We did 20 runs for each different value of *K* with 50,000 iterations following a burn-in period of 50,000. Pairwise comparisons of the 20 solutions of each *K* value were run along with 20 permutations using CLUMPP software [27]. CLUMPP software also outputs a mean of the permuted matrices across replicates after aligning the cluster membership coefficients of these replicate. Finally, the clustering pattern with the highest *H* value was graphically displayed for the selected *K* value using DISTRUCT software [28].

## Results and Discussion

### Mitochondrial COI analysis

**Figure-1:**
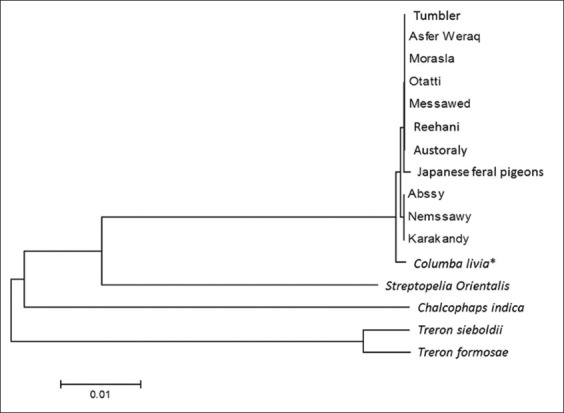
Neighbor-joining tree of mitochondrial *COI* genesequence of 10 Egyptian breeds, Japanese feral pigeons and four wild pigeon species. The sequence for *Columba livia* with an asterisk was retrieved from GenBank (accession number GQ481605). The four wild pigeon species act as outgroup.

We amplified and sequenced 693 bp for one sample of the 10 Egyptian breeds and of Japanese feral pigeons in addition to the four species of wild pigeons for confirmation of the species of Egyptian pigeon. After alignment, there were only one substitution sites among the 10 Egyptian and the Japanese feral pigeons. From the NJ phylogenetic tree (Figure-1), these 11 populations clustered into the same clade with *C. livia* sequence retrieved from GenBank (accession number GQ481605). The branching pattern of other wild species reflected their phylogeny. The low sequence divergence among Egyptian and Japanese feral pigeons together with GenBank sequence of *C. livia* confirms that all of these breeds belong to the same species (*C. livia*) and the mtDNA *COI* sequence divergence is more suited for the analysis of among species divergence than within species divergence [13].

### Microsatellite marker polymorphisms and population diversity

Across the 10 studied Egyptian populations, the number of alleles per locus ranged from 3 (*PG6*) to 19 (*CliµD01*), and the average number of alleles observed was 9.091. Locus *PG6* had the lowest values for both of *H_E_* (0.371) and *PIC* (0.364), whereas locus *CliµD01* had the highest values for *H_E_* (0.731) and *PIC* (0.806), and locus *PG2* recorded the highest *H_O_* (0.727) as shown in Table-2. Locus *PG6* showed lower polymorphism not only in our study but also in previous studies [10,20]. Across the 11 loci, the average numbers of alleles expected and observed heterozygosity in addition to *F_IS_* for each population are shown in Table-3. The lowest value of expected heterozygosity (0.373) was obtained for the Reehani breed, and the highest value (0.706) was found for Morasla breed. The overall expected heterozygosity of Egyptian native pigeons was 0.548. Genetic diversity among studied pigeon breeds showed comparable values with those described by Ramadan *et al*. [10], Bigi *et al*. [11] and Biała *et al*. [12] who studied Egyptian, Polish, and Italian pigeon populations, respectively.

**Table-2:** Observed (N_A_) and effective (N_e_) number of alleles, polymorphism information content (PIC), observed (H_O_), and expected (H_E_) heterozygosities and Fstatistics (F_IS_, FST, and FIT) across the 10 Egyptian populations.

Locus	N_A_±SD	N_e_±SD	H_o_±SD	H_E_±SD	PIC±SD	F_IS_±SD	F_IT_±SD	F_ST_±SD	HWE
CliµD17	6	2.351	0.531	0.542	0.626	-0.011	0.181	0.189	ns
CliµT17	9	2.663	0.635	0.602	0.743	-0.089	0.177	0.245	ns
CliµD16	15	2.257	0.387	0.499	0.531	0.201	0.292	0.114	* * *
CliµD32	11	2.680	0.425	0.492	0.695	0.108	0.381	0.306	*
CliµT13	10	2.943	0.551	0.619	0.781	0.081	0.326	0.266	ns
CliµD01	19	4.164	0.669	0.731	0.806	0.055	0.202	0.155	*
PG1	5	2.307	0.461	0.502	0.655	0.051	0.338	0.303	ns
PG2	10	3.227	0.727	0.694	0.796	-0.081	0.099	0.166	ns
PG4	5	1.979	0.399	0.486	0.651	0.153	0.423	0.318	**
PG6	3	1.663	0.399	0.371	0.364	-0.108	0.038	0.133	ns
PG7	7	2.087	0.453	0.491	0.604	0.050	0.328	0.293	ns
Mean	9.091±0.706	2.575±0.187	0.512±0.055	0.548±0.036	0.659±0.016	0.037±0.043	0.253±0.104	0.226±0.074	
Total mean	10.000±0.707	2.756±0.122	0.535±0.050	0.569±0.028	0.686±0.007	0.068±0.056	0.269±0.093	0.215±0.052	

^a^Total mean includes Japanese feral pigeons in addition to the 10 Egyptian breeds.

As a measure of deviation from Hardy-Weinberg equilibrium, the *F*_IS_ value was calculated and found to range from −0.216 (Asfer Weraq) to 0.194 (Messawed) with a mean 0.031. The inbreeding coefficients (*F_IS_*) were positive for all investigated population except Asfer Weraq and Halaby Asfer which showed negative *F_IS_* values. The higher values for inbreeding coefficient might indicate a departure from random mating, which could be expected for breeding flocks due to artificial selection of mating pairs. There were 16 breeds specific alleles observed among the 11 populations. The number of breed-specific alleles ranged from 0 (Asfer Weraq, Reehani, and Otatti) to 5 (Morasla) as shown in Table-3.

**Table-3:** Mean observed (MN_A_), private alleles, effective (N_e_) number of alleles, observed (H_O_), and expected (H_E_) heterozygosities and fixation coefficient of an individual within a subpopulation (F_IS_) per population.

Population	n total	n male	n female	MNA	Private alleles	N_e_	H_o_	H_e_	FIS	HWE
Mean of Egyptian populations	17.9	10.3	7.6	4.409±1.215	2.286±1.380	2.575±0.630	0.512±0.126	0.548±0.121	0.031±0.121	
Asfer Weraq	14	8	6	2.636	0	1.985	0.503	0.430	−0.216	ns
Austoraly	20	14	6	5.273	3	2.723	0.614	0.625	0.001	ns
Reehani	21	11	10	3.273	0	1.761	0.347	0.373	0.018	ns
Messawed	17	12	5	4.091	1	2.017	0.307	0.420	0.194	* * *
Nemssawy Asfer	27	11	16	3.000	2	1.919	0.359	0.428	0.143	ns
Otatti	12	7	5	4.182	0	2.768	0.595	0.629	0.000	ns
Morasla	17	14	3	6.273	5	3.522	0.618	0.706	0.106	*
Egyptian Tumbler	22	10	12	5.727	1	3.007	0.582	0.646	0.090	* * *
Halaby Asfer	10	4	6	4.364	2	2.652	0.618	0.588	−0.104	ns
Karakandy	19	12	7	5.273	2	3.393	0.580	0.637	0.074	ns
Japanese feral pigeons	30	13	17	7.364	10	4.563	0.758	0.773	0.004	ns
Total mean	19	10.545	8.455	4.678±1.456	3.250±3.012	2.755±0.846	0.535±0.140	0.569±0.133	0.028±0.115	

Genetic differentiation across the 11 studied populations was examined by fixation indices(*F_IS_*, *F_IT_*, and *F_ST_*) for each locus (Table-2). The *F_ST_* coefficient which indicates fixation coefficients of subpopulations within the total population for the 11 loci varied from 0.318 (*PG4*) to 0.114 (*Cliµd16*), with a relatively high mean 0.226, which indicate that there is a genetic differentiation among the 10 populations. This means that about 22.6% of the total genetic variation is due to population’s differences, while the remaining 77.4% is due to differences among individuals. This *F_ST_* value is comparable with those of Ramadan *et al*. [10] who reported the value of 0.203 for Egyptian pigeons and Bigi *et al*. [11] who reported the value of 0.214 for Italian pigeons. However, the *F_ST_* of our study was higher than the value of 0.147 reported by Biała *et al*. [12] among Polish pigeons.

The *F_IT_* coefficient which indicates global deficit of heterozygote across populations amounted to 25.3% (Table-2). For the *F_IS_* coefficient, positivevalues of *F_IS_* indicate deficit of observed heterozygosity; however, negative values might indicate an excess of heterozygous genotypes comparing to the expected value. In our study, the relatively high averageof *F_IS_* (0.037) in addition to seven loci (*CliµD16, CliµD32, CliµT13,CliµD01*, *PG1, PG4*, and *PG7*) recorded a deficiency of heterozygosity; this might indicate that these six loci are under selection for some favorable morphological or productive characteristics.

### Genetic relationship and population structure

**Figure-2:**
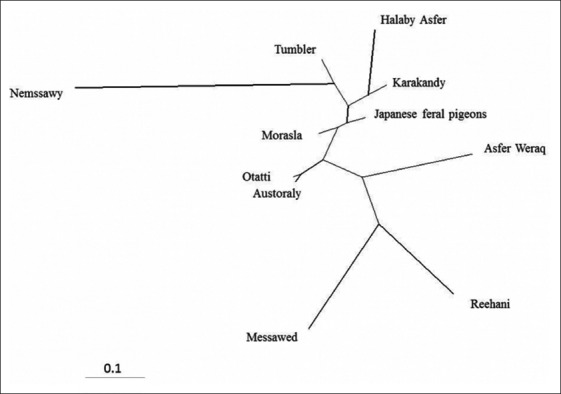
Neighbor-joining tree of 10 Egyptian pigeon breeds and Japanese feral pigeons by 11 microsatellite markers.

**Figure-3:**
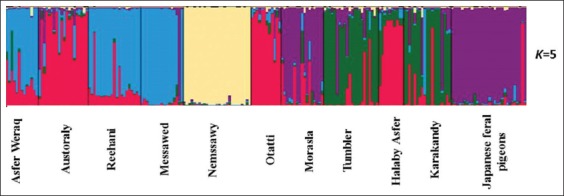
Structure clustering of the 10 Egyptian breeds and Japanese feral pigeons obtained for *K*=5.

The pairwise Reynolds’s genetic distance between the 11 studied pigeon populations recorded lower values between Otatti and Austoraly (0.025) and between Morasla and Japanese feral pigeons (0.054). Similarly, the genetic differentiation indicated by pairwise *F_ST_* values was the lowest in Otatti–Austoraly (0.029) and in Morasla–Japanese feral pigeons (0.039) as shown in Table-4. These results are supported by clustering pattern either by the neighbor-joining phylogenetic tree (Figure-2) or by a Bayesian clustering of STRUCTURE with the admixture method (Figure-3). The tree topology showed close relationship between Otatti and Austoraly and also between Morasla and Japanese feral pigeon populations. At *K*= 5 where the 11 studied pigeon populations showed the most probable structure clustering, Otatti and Austoraly populations were clustered together, and also Morasla and Japanese feral pigeon populations were also clustered together forming admixed mosaic clusters (Figure-3). Nemssawy was assigned independently into its respective clusters; moreover, it showed the highest values for both of Reynolds’s genetic distance and pairwise *F_ST_* with other breeds such as Asfer Weraq, Reehani, and Messawed indicating the uniqueness of Nemssawy breed. Nemssawy is considered as ornamental breed characterized by long and soft feathers around the head and neck. This selective interest for morphological characteristics might explain the uniqueness of Nemssawy breed. The clustering pattern of remaining breeds might be explained by their history and origin.

**Table-4:** Reynolds’s genetic distance (D_A_: above diagonal) and pairwise F_ST_ (below diagonal) estimates for the 11 microsatellite loci between the 11 studied pigeon populations.

Population	Asfer Weraq	Austoraly	Reehani	Messawed	Nemssawy Asfer	Otatti	Morasla	Tumbler	Halaby Asfer	Karakandy	Japanese feral pigeons
Asfer Weraq		0.223	0.239	0.348	0.607	0.210	0.244	0.317	0.222	0.238	0.233
Austoraly	0.136		0.206	0.260	0.442	0.025	0.097	0.149	0.171	0.149	0.116
Reehani	0.111	0.113		0.238	0.642	0.217	0.261	0.349	0.335	0.327	0.258
Messawed	0.179	0.139	0.106		0.668	0.284	0.243	0.377	0.340	0.338	0.236
Nemssawy Asfer	0.297	0.218	0.302	0.327		0.415	0.344	0.315	0.425	0.342	0.297
Otatti	0.131	0.029	0.117	0.152	0.192		0.109	0.149	0.180	0.143	0.120
Morasla	0.147	0.063	0.135	0.136	0.168	0.074		0.113	0.139	0.106	0.054
Egyptian Tumbler	0.181	0.085	0.177	0.199	0.158	0.091	0.068		0.170	0.084	0.106
Halaby Asfer	0.132	0.104	0.156	0.175	0.197	0.109	0.088	0.097		0.067	0.143
Karakandy	0.140	0.091	0.157	0.182	0.166	0.091	0.067	0.043	0.056		0.099
Japanese feral pigeons	0.149	0.069	0.144	0.140	0.159	0.078	0.039	0.062	0.095	0.065	

The close relationship between Otatti and Austoraly might be attributed to that Austoraly is a synthetic breed originated from crossing between Safi with Zagel and Otatti breeds [29]. The close relationship between Morasla and Japanese feral population could be attributed to that Morasla breed might be developed from free-living feral populations, or Morasla might contribute to the genetic profile of feral populations. Morasla is an Arabic name means messenger or carrier pigeons. The messenger and racing pigeons had been established by Belgians through the crossing of free-living pigeons with several types of domestic pigeons for the improvement of fitness and homing ability. Previously, Stringham *et al*. [30] found little genetic differentiation between racing homer and feral pigeons indicated by lower values for both of genetic distance and pairwise *F_ST_*. Moreover, they found a close relationship between European free-living populations with a former racing breed called Modena. Modena pigeon breed was developed in Italy since 2000 years ago [31]. They suggested that either Modena racing breed was developed from European free-living populations or racing populations contributed to the genetic constitution of local feral populations, especially some pigeon breeders report that up to 20% of their racing birds that start racing competitions do not return again and become feral. In the same way, Ramadan *et al*. [10] found a close relationship between Egyptian Zagel breed and Japanese racing pigeons and attributed this close relationship to that they might have a common ancestor. Genetic studies including many feral and racing populations from different localities all over the world might help us to solve this controversial finding and to understand whether this close genetic relationship between racing and feral populations is occurred sporadically or as we expect to be everywhere.

### Cross-species amplification by microsatellite markers

We investigated the applicability of 11 microsatellite markers of *C. livia* species across eight wild pigeon species by means of cross-species amplification(Table-5). We obtained 170 alleles by cross-amplification of 11 markers among eight wild species. *CliµD32* and *PG6* showed successful cross-species amplification among all the studied wild pigeon species. On the contrary, *PG1* could be amplified only across oriental turtle dove. 10 of 11 studied loci were successfully amplified in the oriental turtle dove, and all of these loci were polymorphic. Loci showed that monomorphism across our wild pigeon species might show polymorphism if a larger sample size was assessed. Pruett *et al*. [32] found that eight microsatellite markers of common raven (*Corvus corax*) were amplified and showed polymorphism across six of the seven studied Corvidae species.

**Table-5:** Number of alleles of 11 microsatellite loci in nine Columbidae species.

Species	n	Number of alleles per locus										

		CliµD17	CliµT17	CliµD16	CliµD32	CliTµ13	CliµD01	PG1	PG2	PG4	PG6	PG7
Oriental turtle dove (Streptopelia orientalis)	15	9	7	2	4	-	4	6	8	8	2	3
Whitebellied green pigeon (Treron sieboldii)	9	4	6	5	1	-	8	-	-	6	3	3
Emerald dove (Chalcophaps indica)	4	-	2	4	4	-	2	-	-	-	2	1
Whistling green pigeon (Treron formosae)	3	1	4	2	1	3	3	-	-	2	2	-
Blue crowned pigeon (Goura cristata)	2	-	4	-	1	-	2	-	-	2	1	-
Japanese wood pigeon (Columba janthina janthina)	2	-	2	-	2	2	2	-	2	2	2	2
Victoria crowned pigeon (Goura victoria)	1	-	1	-	2	-	-	-	-	2	1	-
Pied imperial pigeon (Ducula bicolor)	1	-	-	-	2	-	-	-	2	2	2	2

- =Individuals failed to amplify at a locus

## Conclusion

We confirm the applicability of microsatellite markers among Egyptian domestic pigeons and across wild pigeon species by means of cross-species amplification. Relatively reliable diversity results could be obtained even with a small number of polymorphic microsatellites, as shown in our study and confirmed by other similar studies [12]. The information from this study should be useful for genetic characterization and for developing conservation programs of this agriculturally and commercially important species.

## Authors’ Contributions

SR and MI planned and designed the study. AD, OE, and AMN collected the feather samples and provided the help for statistical analysis. SR performed microsatellite genotyping and drafted the manuscript under the guidance of MI. ME and AMN provided the help for the data analyzed and interpretation of laboratory results. All authors participated in draft and revision of the manuscript. All authors read and approved the final manuscript.
